# Scalp microbiome in male androgenetic alopecia: *16S* rRNA sequencing-based clinical characterization, mouse model validation, and effects on hair follicle cells

**DOI:** 10.3389/fcimb.2026.1878609

**Published:** 2026-07-01

**Authors:** Lingyi Zhang, Menghui Qin, Danyu Li, Kun Wang, Tengfei Wang, Yaoyao Zhou, Xin Yao, Yingying Tian, Mengru Pang

**Affiliations:** 1 Department of Burns and Plastic Surgery, The Affiliated Hospital of Guizhou Medical University, Guiyang, China; 2 The Key Laboratory of Environmental Pollution Monitoring and Disease Control, Ministry of Education, School of Public Health, Guizhou Medical University, Guiyang, China

**Keywords:** androgenetic alopecia, inflammation, microbial dysbiosis, phenol-soluble modulins, scalp microbiota

## Abstract

**Introduction:**

Persistent microinflammation in androgenetic alopecia (AGA) may contribute to hair follicle miniaturization, but whether scalp microbial dysbiosis serves as its trigger remains unclear. This study integrated clinical samples, an animal model, and *in vitro* experiments to investigate the scalp microbiome in AGA.

**Methods:**

Scalp microbial samples were collected from four regions (frontal, vertex, temporal, occipital) of 12 AGA patients and 12 healthy controls (96 samples in total) and analyzed by 16S rRNA gene sequencing. An AGA mouse model was established using testosterone propionate to evaluate histopathology and skin microbiota. The effects of *Staphylococcus epidermidis*-derived phenol-soluble modulins PSMγ and PSMδ on human dermal papilla cell (HDPC) proliferation were assessed, and the effect of PSMδ on cell migration was also examined.

**Results:**

AGA patients exhibited elevated overall scalp microbial richness and diversity, with a marked decrease in *Staphylococcus* abundance that was most pronounced in the frontal and vertex regions. The mouse model also displayed significant restructuring of the skin microbiota; however, *Staphylococcus* showed marked enrichment—a direction opposite to that in humans. Together, these findings indicate that aberrant *Staphylococcus* abundance serves as a sensitive bio-indicator of the AGA pathological state. PSMδ significantly promoted HDPC proliferation in a time- and concentration-dependent manner, demonstrating a wider effective concentration window and milder action, and it also significantly enhanced cell migration.

**Discussion:**

The dysregulation of *Staphylococcus* abundance is a key feature of scalp microbial dysbiosis in AGA. PSMδ possesses dual potential in modulating hair follicle cell activity and maintaining microecological homeostasis, providing new insights for microbiome-targeted interventions in AGA.

## 1 Introduction

Androgenetic alopecia (AGA) is the most common form of progressive hair loss worldwide. Over half of Caucasian men are affected to varying degrees by the age of 50 (Asfour et al., 2023), and women are also commonly affected. The incidence rises with age and shows a clear trend toward earlier onset. AGA is not merely a cosmetic concern; its detrimental impact on mental health and social functioning is increasingly recognized. Although hair loss itself typically does not cause significant somatic discomfort, the progressive change in appearance can lead to substantial psychological distress. Numerous studies have demonstrated that the prevalence of anxiety and depression is markedly higher in AGA patients than in healthy controls, frequently accompanied by diminished self-esteem, social avoidance behaviors, and concerns regarding professional appearance (Aukerman and Jafferany, 2023). AGA, as the most common form of hair loss, has long been underestimated in terms of its disease burden in clinical practice (Han et al., 2012). Therefore, further elucidating the pathogenesis of AGA and exploring novel intervention strategies not only holds biological significance for hair regeneration but also carries important clinical value for improving patients’ mental health and quality of life.Current clinical treatment primarily focuses on modulating androgen signaling pathways and promoting hair growth. Although the existing therapeutic system is relatively comprehensive, some patients still face limitations such as inadequate efficacy, insufficient response, or intolerance to adverse drug reactions (Rossi et al., 2012; Ganzer et al., 2015; Hirshburg et al., 2016; Wang et al., 2023a). Notably, persistent low-grade inflammation is commonly present on the scalp of AGA patients, and its severity is positively correlated with the degree of hair follicle miniaturization (Heymann, 2022; Plante et al., 2022). However, current therapeutic strategies pay insufficient attention to this microinflammation, and the specific triggers of this microinflammation remain undefined.

Different anatomical sites of the human body harbor highly site-specific microbial communities, and disruption of their homeostasis has been implicated in the pathogenesis of various skin diseases, including atopic dermatitis (Gallo and Horswill, 2024), acne (Podwojniak et al., 2025), psoriasis (Li et al., 2025), and even cutaneous neoplasms (Voigt et al., 2022; Charbel et al., 2026; Nakatsuji et al., 2018). Furthermore, commensal microbiota play a critical regulatory role in hair follicle regeneration: germ-free mice exhibit an approximately 18-fold reduction in wound-induced hair follicle neogenesis compared with conventionally raised mice (Wang et al., 2021; Wang et al., 2023b), indicating that an intact microbial community is essential for maintaining hair follicle regenerative potential. The scalp, with its dense distribution of sweat glands, abundant sebaceous glands, and relatively high humidity, provides a microenvironment far more conducive to microbial colonization than other skin sites, and its microbial load is significantly higher (Paul et al., 2025).Under normal conditions, the scalp microbiome is dominated by bacteria, primarily *Cutibacterium* and *Staphylococcus*, and fungi, predominantly *Malassezia* (Byrd et al., 2018). Disruption of this microbial homeostasis, with overgrowth of specific microorganisms, can give rise to various scalp disorders: for instance, *Malassezia* overgrowth is closely associated with seborrheic dermatitis, and the predominance of *Staphylococcus aureus* is linked to folliculitis (Saxena et al., 2018). Scalp microbial dysbiosis has been shown to be associated with scalp disorders such as alopecia areata, seborrheic dermatitis, and folliculitis (Shah et al., 2024), and twin studies suggest that exogenous factors also contribute to the development of AGA (Koyama et al., 2013). Nevertheless, systematic investigations into the region-specific distribution patterns of the scalp microbiome in AGA patients, and whether microbial dysbiosis participates in triggering microinflammation and subsequently affects hair follicle cell function, remain lacking.

This study aims to preliminarily characterize the structural features and regional heterogeneity of the scalp microbiome in AGA patients through integrated clinical sample sequencing, animal model validation, and *in vitro* cellular experiments, and to explore potential associations among microbial dysbiosis, microinflammation, and hair follicle cell function. These findings are expected to provide foundational data and new insights for future screening of AGA-related microbial biomarkers and the exploration of adjuvant intervention strategies targeting the scalp microecosystem.

## 2 Materials and methods

### 2.1 Subject recruitment and ethical oversight

Twelve male AGA patients (Hamilton−Norwood grade II–V, diagnosed per the Chinese Guidelines for the Diagnosis and Treatment of Androgenetic Alopecia [2023]) and 12 age−matched healthy male controls were enrolled from the Affiliated Hospital of Guizhou Medical University (February–May 2025). All subjects were aged 18–40 years with BMI 18.5–24.0 kg/m². Only male subjects were enrolled to minimize the confounding effects of hormonal milieu and hair loss pattern differences between sexes, thereby enabling a more interpretable regional microbiome analysis with a limited sample size. The scalp microbiome characteristics of female AGA patients warrant dedicated investigation in future studies. Detailed eligibility criteria and diagnostic procedures are provided in the **Supplementary Material**. The study was approved by the Clinical Ethics Committee (Non-Drug Research) of Guizhou Medical University (Approval No. 2024-Lunshen-No. 478, December 31, 2024) and conducted in accordance with the 2024 Declaration of Helsinki. All participants provided written informed consent.

### 2.2 Skin swab collection

Sterile cotton swabs pre−moistened in sterile saline were used to sample four scalp regions (frontal, vertex, temporal, occipital; 16 cm² per site). Swab heads were aseptically collected into sterile tubes and stored at −80 °C. Dorsal skin swabs from mice were collected using the same protocol.

### 2.3 DNA extraction, *16S* rRNA gene amplification, and bioinformatic processing

Total genomic DNA was extracted from 96 scalp swabs using the TIANamp Swab DNA Kit (Tiangen, China). The V3–V4 region of the *16S* rRNA gene was amplified with primers 341F/806R, purified, and sequenced on an Illumina MiSeq platform (2 × 300 bp). Raw data were processed with FLASH and fastp, chimeras were removed against SILVA (v138.1), and ASVs were inferred with DADA2 in QIIME2 (v2022.2). Taxonomy was assigned against SILVA 138.1.

### 2.4 Microbiota diversity and statistical analysis

Alpha diversity (Shannon, Simpson, Observed ASVs, Chao1; at the ASV level) was compared between groups using multivariate linear regression and ANOVA. Beta diversity was assessed with unweighted UniFrac distance and PERMANOVA (Adonis, 999 permutations). PCoA with 95% confidence ellipses was used for ordination. Microbiome-specific analyses were performed in R (v4.5.2; vegan, phyloseq). All other statistical analyses were performed using SPSS 30.0, with GraphPad Prism 10.0 used for figure generation. Data are presented as mean ± SEM. After testing for normality (Shapiro−Wilk) and homogeneity of variance (Levene), parametric or non−parametric tests were applied as appropriate. Multiple comparisons were corrected using Bonferroni or Benjamini−Hochberg procedures. *P* < 0.05 was considered significant.

### 2.5 Experimental animals and androgenetic alopecia mouse model

Twelve male C57BL/6 mice (4–5 weeks old) were randomized into control and AGA groups (n = 6 each). Mice were housed under SPF conditions. AGA was induced by daily subcutaneous injection of testosterone propionate (5 mg/kg in corn oil) for 8 weeks; controls received corn oil vehicle. The phenotype was confirmed by visibly sparse dorsal hair.

### 2.6 Histology and immunofluorescence staining

Dorsal skin was fixed in 4% paraformaldehyde, paraffin−embedded, and sectioned at 5 μm. H&E staining followed standard protocols; histomorphometry (epidermal/dermal thickness, hair follicle density) was performed with ImageJ. Multiplex immunofluorescence used TSA with anti−F4/80, anti−CD3, and anti−CD14 antibodies. Nuclei were counterstained with DAPI. Images were acquired on a Nikon Eclipse C1 microscope and analyzed via whole-slide scanning.

### 2.7 Preparation of phenol−soluble modulins and cell−based assays

PSMγ and PSMδ (≥95% purity by HPLC) were synthesized by Fmoc solid−phase peptide synthesis. Immortalized human dermal papilla cells (iCell-0163a), purchased from iCell Bioscience Inc. (Shanghai, China), were cultured in serum−free hFIB Max medium. Proliferation was assessed with the CCK−8 assay after 12–72 h exposure to PSMγ or PSMδ (1–128 μM). Migration was evaluated by scratch wound healing assay with PSMδ (0–9 μM) and quantified using ImageJ.

## 3 Results

### 3.1 Sequencing depth and ASV derivation

Sequencing generated a total of 4,866,807 and 4,822,150 raw V3-V4 *16S* rRNA reads from the healthy (n=12) and AGA (n=12) cohorts, respectively, with comparable average depths (103,551 ± 11,254 vs. 102,599 ± 12,568 reads per sample). Processing with DADA2 yielded 11,655 high-quality, non-redundant Amplicon Sequence Variants (ASVs) for downstream analysis. All ASVs were retained without applying a low-abundance filter, as each represented a unique sequence at 100% similarity. Per-sample ASV counts averaged 892 (range: 576–1,189; SD: 127).

### 3.2 Phylum-level compositional differences in scalp bacterial communities between healthy controls and AGA patients

**Figure 1:**
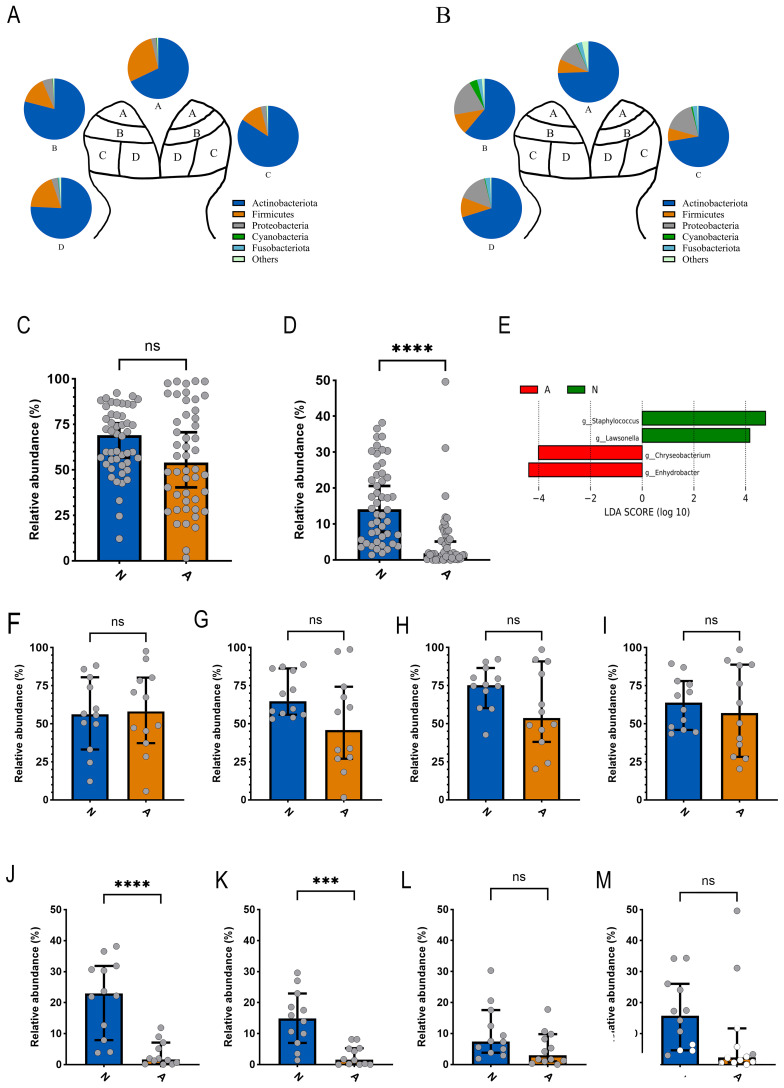
Regional variations in scalp bacterial community composition. **(A, B)** Pie charts showing the regional distribution of the top five most abundant phyla across the frontal (F), vertex (V), temporal (T), and occipital (O) scalp regions in healthy controls **(A)** and AGA patients **(B)**. **(C, D)** Relative abundances of *Cutibacterium* **(C)** and *Staphylococcus* **(D)** at the genus level between healthy (N) and AGA (A) groups at the whole-scalp level. **(E)** Linear discriminant analysis effect size (LEfSe) identified *Staphylococcus* as significantly enriched in the healthy group (N) (LDA score > 2). **(F–I)** Region-specific comparisons of the relative abundance of *Cutibacterium* between the N and A groups in the frontal **(F)**, vertex **(G)**, temporal **(H)**, and occipital **(I)** regions. **(J–M)** Region-specific comparisons of the relative abundance of *Staphylococcus* between the N and A groups in the frontal **(J)**, vertex **(K)**, temporal **(L)**, and occipital **(M)** regions.Data are presented as mean ± s.d. N, healthy group; A, androgenetic alopecia group. Statistical significance was determined using a two-tailed Mann-Whitney U test (*P*: ***< 0.001; ****< 0.0001; ns, not significant).

At the phylum level, comparable phylogenetic breadth was observed between the two groups (35 phyla in healthy controls; 38 in AGA). Both groups were dominated by Actinobacteriota, Firmicutes, Proteobacteria, Cyanobacteria, and Fusobacteriota. However, Cyanobacteria sequences were primarily attributed to chloroplast contamination from environmental sources and were excluded from further analysis. Quantitative analysis revealed consistent compositional shifts across all four scalp regions in AGA patients relative to healthy controls (**Figures 1A, B**; **Table 1**). Specifically, Firmicutes abundance was consistently lower in AGA samples across all regions, whereas Proteobacteria abundance was consistently higher. These reciprocal shifts were most pronounced in the frontal and vertex regions (**Table 1**).

**Table 1:** Relative abundance (%) of dominant bacterial phyla in different scalp regions from healthy controls and patients with androgenetic alopecia (AGA).

Group	Scalp region	Actinobacteriota	Firmicutes	Proteobacteria	Cyanobacteria	Fusobacteriota
Healthy Controls (N = 12)	Frontal(A)	67.87	28.36	2.75	0.33	0.02
	Vertex(B)	79.02	14.49	5.45	0.23	0.05
	Temporal(C)	84.19	11.84	2.90	0.12	0.09
	Occipital(D)	75.76	19.02	3.36	0.21	0.65
AGA Patients (N = 12)	Frontal(A)	74.42	7.31	11.66	0.45	2.71
	Vertex(B)	61.10	11.10	19.53	4.24	2.51
	Temporal(C)	72.37	7.00	16.70	0.77	2.41
	Occipital(D)	70.17	10.63	15.40	0.43	2.62

A total of 35 and 38 bacterial phyla were identified in the healthy control and AGA groups, respectively. The composition of the dominant phyla was identical between groups, but their relative abundances differed significantly across scalp regions.

### 3.3 Region-specific depletion of *Staphylococcu*s in AGA

**Figure 2:**
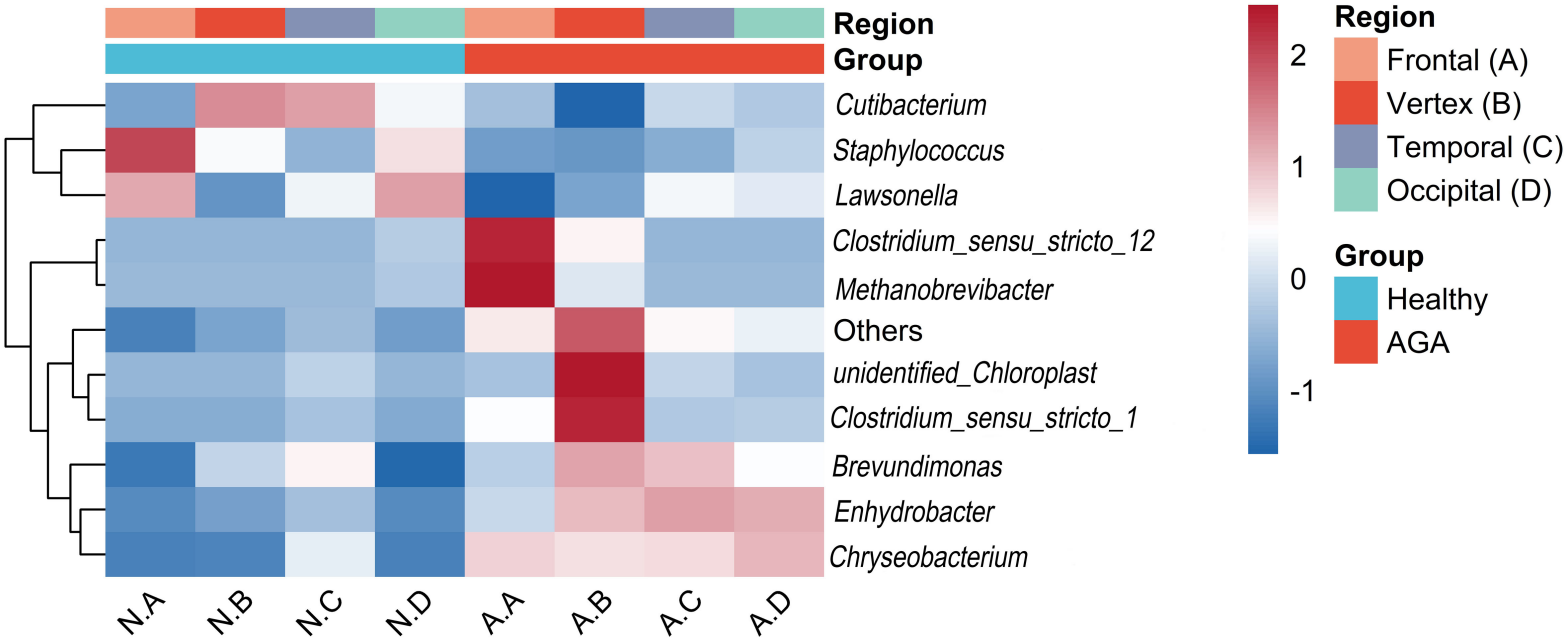
Heatmap of genus-level scalp microbiome composition in healthy controls and AGA patients across four scalp regions. patients (A). Relative abundances were Z-score normalized by row. Rows (genera) were clustered using hierarchical clustering with Euclidean distance and complete linkage method. Columns (sample groups) were not clustered and are displayed in the following order: healthy frontal (N.A), healthy vertex (N.B), healthy temporal (N.C), healthy occipital (N.D), AGA frontal (A.A), AGA vertex (A.B), AGA temporal (A.C), and AGA occipital (A.D). The color scale represents Z-scores, with blue indicating low relative abundance, white indicating intermediate abundance, and red indicating high relative abundance. The top annotation bars indicate group (Healthy, blue; AGA, red) and scalp region (frontal, light orange; vertex, red; temporal, purple; occipital, teal). Note that Staphylococcus and *Cutibacterium* clustered together as the dominant genera enriched in healthy controls.

Analysis of the two dominant genera, *Cutibacterium* and *Staphylococcus*, revealed contrasting patterns (**Figure 2**). *Cutibacterium* abundance did not differ significantly between the AGA and healthy control groups at the whole-scalp level (P > 0.05; **Figures 1C**; **Table 2**), and no significant differences were observed in any individual scalp region (**Figures 1F–I**; **Table 3**). In contrast, *Staphylococcus* abundance was markedly reduced in AGA patients at the whole-scalp level (*P* < 0.0001; **Figure 1D**; **Table 2**). This reduction was highly region-dependent (**Figures 1J–M**; **Table 2**): the most pronounced depletion was observed in the frontal region, where *Staphylococcus* abundance in AGA patients dropped to approximately 16% of that in healthy controls (mean 3.65% vs. 22.60%, *P* < 0.0001), followed by the vertex region (approximately 37% of controls, 5.57% vs. 14.91%, *P* < 0.001). In contrast, differences in the temporal and occipital regions were not statistically significant (all *P* > 0.05). Differential abundance analysis further identified *Staphylococcus* as significantly enriched in the healthy group (**Figure 1E**). In summary, the scalp microbiota in AGA is characterized by a marked, region-selective loss of *Staphylococcus* that is most pronounced in the anterior scalp, whereas *Cutibacterium* abundance does not differ significantly between groups.

**Table 2:** Overview of bacterial composition at the whole-scalp level.

Metric/group	Healthy controls (n=12)	AGA patients (n=12)
Number of Bacterial Genera Identified	624	798
Number of Bacterial Species Identified	386	592
Cutibacterium (Mean ± SD, %)	65.63 ± 18.53	56.67 ± 27.89
Staphylococcus (Mean ± SD, %)	15.90 ± 10.86	5.15 ± 8.66 ****

The combined relative abundance of *Cutibacterium* and *Staphylococcus* exceeded 60% in both groups. ****P < 0.0001 vs. Healthy Controls (Mann-Whitney U test).

**Table 3:** Regional relative abundance (%) of *Cutibacterium* and *Staphylococcus* on the scalp.

Scalp region	Group	*Cutibacterium* (mean ± SD, %)	*Staphylococcus* (mean ± SD, %)
Frontal (A)	Healthy Controls	55.94 ± 25.05	27.29 ± 21.70
	AGA Patients	58.34 ± 27.51	2.89 ± 3.96 ****
Vertex (B)	Healthy Controls	69.96 ± 14.44	14.83 ± 8.87
	AGA Patients	49.85 ± 31.07	2.89 ± 3.18 ***
Temporal(C)	Healthy Controls	74.00 ± 14.20	10.35 ± 8.52
	AGA Patients	59.30 ± 26.57	5.18 ± 5.46
Occipital(D)	Healthy Controls	63.62 ± 16.83	16.42 ± 11.24
	AGA Patients	59.20 ± 28.74	9.15 ± 15.43

Statistical significance was determined by the Wilcoxon signed-rank test for pairwise comparisons between groups within the same scalp region (n=12 per comparison). ***P < 0.001, ****P < 0.0001 vs. Healthy Controls in the same region. n.s., not significant.

### 3.4 Alpha diversity of the scalp microbiota

**Figure 3:**
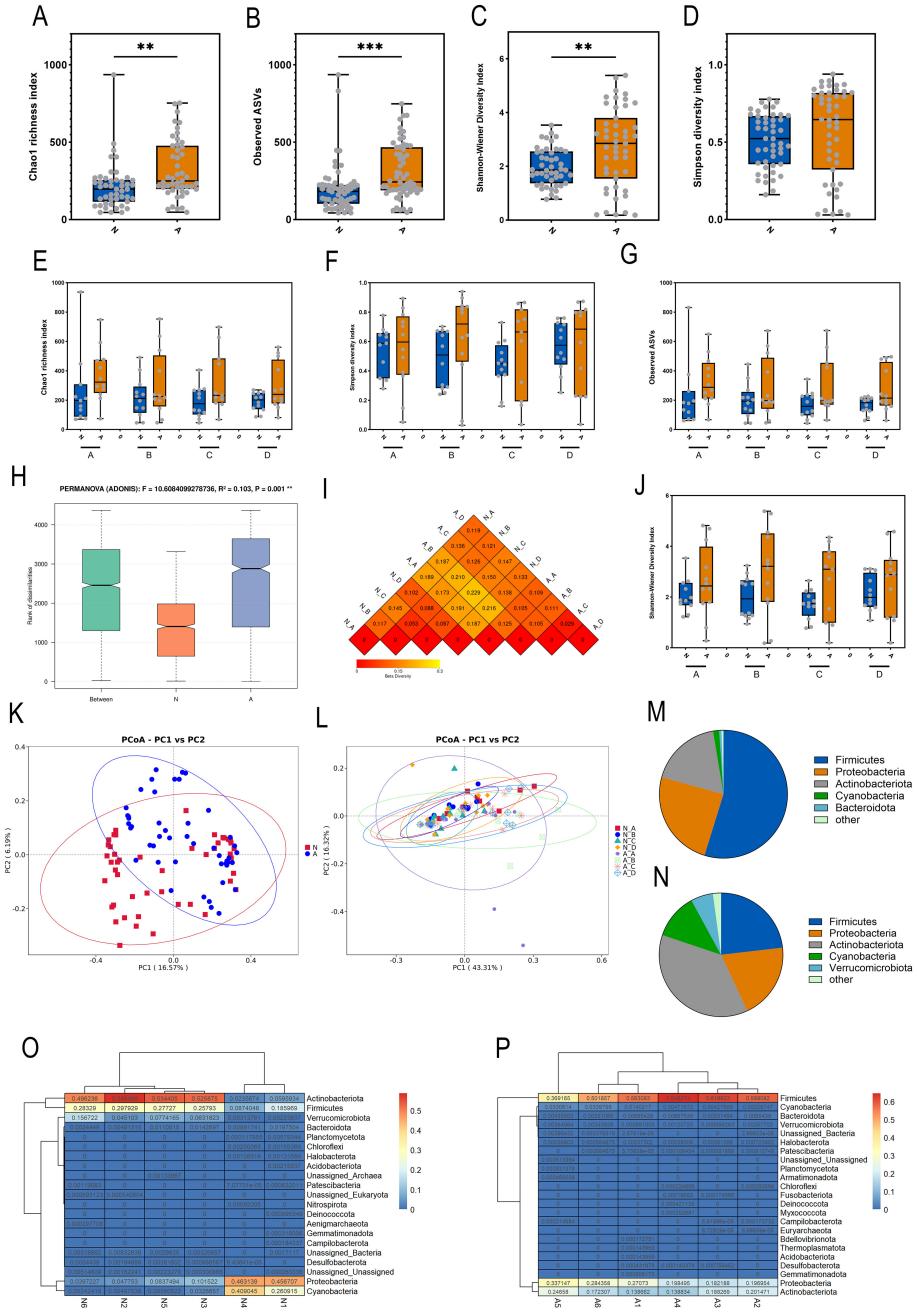
Diversity and composition of scalp bacteria in humans and mice. **(A–D)** Comparisons of α-diversity indices between healthy (N) and AGA (A) groups across the entire scalp: Chao1 index **(A)**, number of observed amplicon sequence variants (ASVs) **(B)**, Shannon index **(C)**, and Simpson index **(D)**. **(E–G, J)** Regional comparisons of α-diversity indices between the N and A groups: Chao1 index **(E)**, Simpson index **(F)**, number of observed ASVs **(G)**, and Shannon index **(J)**. **(H)** Permutational multivariate analysis of variance (PERMANOVA, Adonis) revealed a statistically significant difference in bacterial community structure between N and A groups (R² = 0.103, P = 0.001). **(I)** β-diversity analysis assessed by unweighted UniFrac distance. **(K, L)** Principal coordinate analysis (PCoA) plots based on unweighted UniFrac distance, showing the ordination of bacterial communities in the N and A groups.**(M, N)** Pie charts showing the relative abundance of the top five bacterial phyla in healthy **(M)** and AGA **(N)** mice. **(O, P)** Heatmaps illustrating the taxonomic composition at the phylum level in healthy **(O)** and AGA **(P)** mice. Data are presented as mean ± s.d. N, healthy group; A, androgenetic alopecia group. Statistical significance for α-diversity indices was determined using multivariate linear regression and ANOVA. For other comparisons, a two-tailed Mann-Whitney U test was used (P: **< 0.01; ***< 0.001).

Analysis of alpha diversity at the whole-scalp level revealed significant differences between AGA patients and healthy controls. The Chao1 index was significantly higher in the AGA cohort (P < 0.01; **Figure 3A**), indicating greater estimated species richness. This was corroborated by a significantly higher number of observed ASVs in AGA patients (*P* < 0.001; **Figure 3B**), confirming an increase in actual detected species. The Shannon index was also significantly elevated in the AGA group (P < 0.05; **Figure 3C**), indicating that both species richness and community evenness were enhanced. In contrast, the Simpson index, which places greater weight on dominant species, showed no significant difference between groups (*P* > 0.05; **Figure 3D**), suggesting that the overall dominance structure was not substantially altered in AGA.

When analyzed by individual scalp regions, all alpha diversity indices (Chao1, observed ASVs, Shannon; **Figures 3E–G, J**) showed trends consistent with the whole-scalp analysis, with AGA regions displaying higher mean values. However, none of these regional differences reached statistical significance (all *P* > 0.05), which may be attributable to the reduced sample size per region after stratification.

### 3.5 Beta diversity of the scalp microbiota

Beta diversity was assessed using unweighted UniFrac distances. At the whole-scalp level, a significant difference in microbial community structure was observed between the healthy control (N) and AGA (A) groups, as confirmed by PERMANOVA (R² = 0.103, *P* = 0.001; **Figure 3H**). Principal coordinate analysis (PCoA) showed partial overlap between the two groups, with the AGA group exhibiting lower within-group dispersion than the healthy group (**Figures 3K–L**). This indicates that AGA patients displayed more constrained inter-individual variation, whereas healthy individuals showed greater heterogeneity. The convergence of microbial communities in the AGA group suggests a directional effect of the disease on the scalp microbiome.

Upon stratification by scalp region, within-subgroup dispersion was further reduced in most individual regions compared with the whole-scalp analysis. Among all subgroups, the AGA frontal subgroup showed the lowest dispersion, indicating that the microbial communities in the primary affected region were the most constrained by disease status. Within the same anatomical region, discernible differences were observed between AGA and healthy subgroups, and differences were also noted across different scalp regions.

### 3.6 Microbial dysbiosis in an AGA mouse model

**Figure 4:**
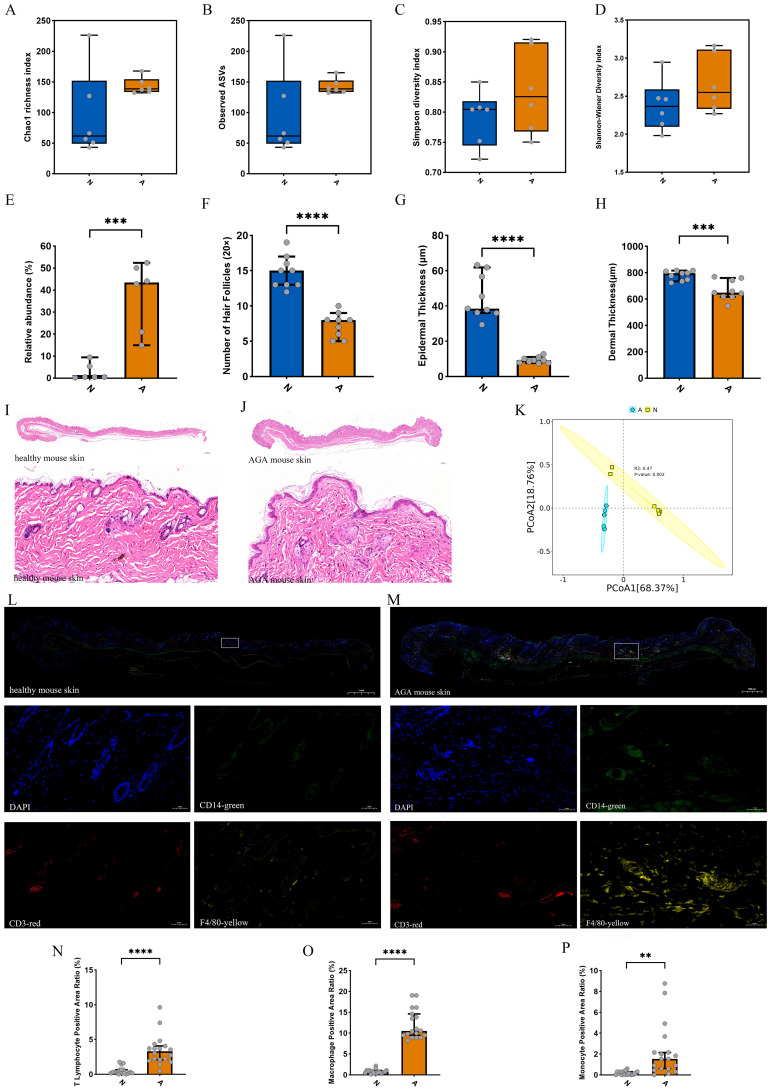
Diversity, histological and immunological traits of mouse skin tissues. **(A–D)** α-diversity indices including Chao1 richness **(A)**, number of observed ASVs **(B)**, Simpson index **(C)**, and Shannon index **(D)** were compared between healthy (N) and AGA (A) mice. **(E)** Relative abundance of *Staphylococcus* at the genus level was compared between groups. **(F–H)** Histomorphometric analyses quantified hair follicle density **(F)**, epidermal thickness **(G)**, and dermal thickness **(H)**. **(I, J)** Representative hematoxylin and eosin (H&E)-stained skin sections from healthy **(I)** and AGA **(J)** mice. **(K)** Principal coordinate analysis (PCoA) based on unweighted UniFrac distance illustrates β-diversity of bacterial community composition. **(L, M)** Immunofluorescence micrographs of skin tissues from healthy **(L)** and AGA **(M)** mice, stained for DAPI (nuclei, blue), CD14^+^ cells (green), CD3^+^ T lymphocytes (red), and F4/80^+^ macrophages (yellow). **(N–P)** Infiltration levels of CD3^+^ T cells **(N)**, CD14^+^ cells **(O)**, and F4/80^+^ macrophages **(P)** were quantified.Data are presented as mean ± s.d. N, healthy mice; A, androgenetic alopecia mice. Statistical significance for α-diversity indices was determined using multivariate linear regression and ANOVA. For other comparisons, a two-tailed Mann-Whitney U test was used (*P*: **< 0.01 ***< 0.001 ****< 0.0001).).

To validate the clinical findings in a controlled experimental setting, we profiled the dorsal skin microbiota in a testosterone propionate-induced AGA mouse model and healthy controls (n = 6 per group). At the phylum level, AGA mice exhibited a pronounced increase in Firmicutes relative to controls (**Figures 3M–P**). Notably, and in striking contrast to the depletion of *Staphylococcus* observed on human AGA scalps, *Staphylococcus* was dramatically enriched in AGA mouse skin (37.57% vs. 2.11% in controls; *P* < 0.001; **Figure 4E**). Detailed taxonomic compositions are provided in **Tables 4**–**6**. Alpha diversity did not differ significantly between the two groups (**Figures 4A–D**). However, beta diversity analysis revealed a significant separation in community structure between AGA and control mice (PERMANOVA, R² = 0.47, *P* = 0.002; **Figure 4K**). These findings indicate that androgenetic alopecia is associated with substantial restructuring of the skin microbiota in both humans and mice, although the direction of change in specific taxa may differ between species.

**Table 4:** Overview of sequencing depth and diversity in dorsal skin microbiota of AGA model and control mice.

Group	Sample size (n)	No. of phyla	No. of genera	No. of species
AGA Model Mice	6	22	261	367
Healthy Control Mice	6	20	248	327

**Table 5:** Relative abundance of major microbial taxa in dorsal skin microbiota of AGA model and control mice.

Taxonomic level	AGA model mice (n=6)	Healthy control mice (n=6)
Phylum Level (Top 3, %)	Firmicutes (54.8%)	Actinobacteriota (37.1%)
	Proteobacteria (24.5%)	Firmicutes (23.2%)
	Actinobacteriota (18.1%)	Proteobacteria (19.9%)
Genus Level (Top 5, %)	Staphylococcus (37.6%)	Pseudomonas (13.7%)
	Pseudomonas (18.4%)	Marinibacillus (9.5%)
	Bifidobacterium (5.7%)	Akkermansia (5.8%)
	Corynebacterium (5.5%)	Bacillus (4.3%)
	Lactobacillus (3.6%)	Pseudarthrobacter (3.8%)

**Table 6:** Statistical comparison of key differentially abundant microbial taxa between AGA model and control mice.

Key taxon	AGA model mice	Control mice	significance
Firmicutes (Phylum)	54.79	23.16	*
Staphylococcus (Genus)	37.57	2.11	**

Intergroup comparisons were performed using two-tailed Mann-Whitney U tests. *P < 0.05, **P < 0.01. Rel. Abundance, Relative Abundance.

### 3.7 Histopathological analysis of hair follicle morphology in mouse skin

H&E staining of dorsal skin sections revealed significant morphological alterations in the AGA group compared with controls (**Figures 4I, J**). The epidermis of control mice was uniformly thick with well-organized, stratified keratinocytes, whereas the AGA group exhibited epidermal thinning (*P* < 0.001; **Figure 4G**), irregular thickness, and loss of cellular organization. Control skin contained numerous, large, and morphologically intact hair follicles with distinct bulbs, while AGA skin showed a significant reduction in hair follicle density (*P* < 0.001; **Figure 4F**), with the remaining follicles predominantly miniaturized, featuring shrunken bulbs and reduced overall size. The dermal layer was significantly thinner in AGA mice (*P* < 0.001; **Figure 4H**), and at higher magnification, dermal collagen fibers appeared disorganized and densely packed, a pattern suggestive of dermal remodeling.

### 3.8 Enhanced immune cell infiltration in AGA mouse skin

To determine whether the histological alterations observed in AGA mice were accompanied by inflammatory changes, we performed multiplex immunofluorescence staining on dorsal skin sections. AGA mice exhibited increased infiltration of immune cells compared with healthy controls (**Figures 4L–P**). The fluorescence signal intensity of macrophages (F4/80^+^) was significantly elevated in the AGA group (*P* < 0.05), while signals for T lymphocytes (CD3^+^) and CD14^+^ cells were markedly stronger (*P* < 0.001). These findings indicate the presence of a robust inflammatory response in the skin of AGA model mice.

### 3.9 PSMδ exhibits superior and more sustained pro-proliferative activity on HDPCs

**Figure 5:**
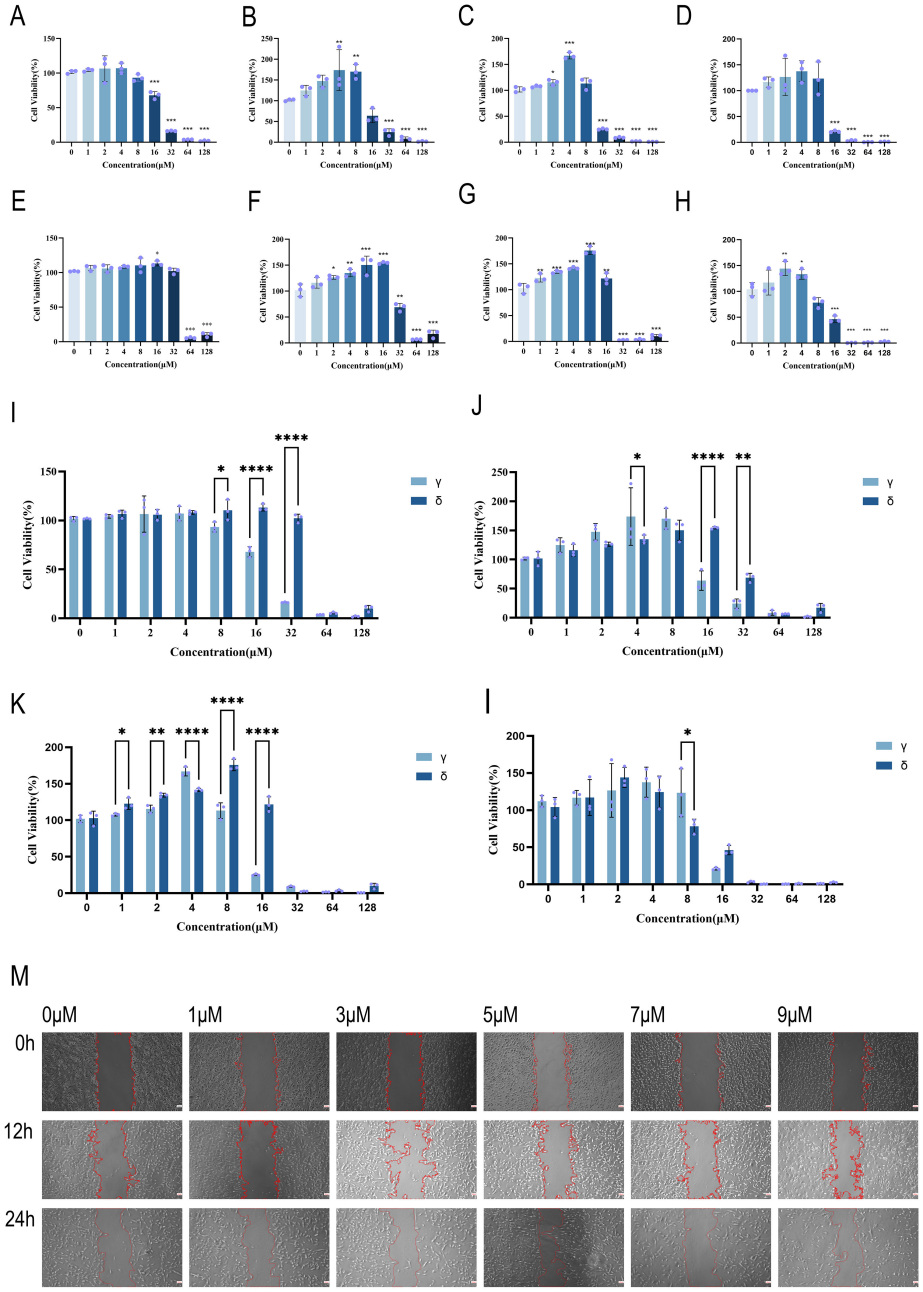
Effects of PSMγ and PSMδ on HDPC proliferation and migration. **(A–D)** Proliferation rates of HDPCs following treatment with PSMγ at 12 h **(A)**, 24 h **(B)**, 48 h **(C)**, and 72 h **(D)**. **(E–H)** Proliferation rates of HDPCs following treatment with PSMδ at 12 h **(E)**, 24 h **(F)**, 48 h **(G)**, and 72 h **(H)**. **(I–L)** Direct comparison of the proliferation rates between PSMγ- and PSMδ-treated HDPCs at the same concentration at 12 h **(I)**, 24 h **(J)**, 48 h **(K)**, and 72 h **(L)**. **(M)** Representative images of scratch wound healing assay showing HDPC migration at 0, 12, and 24 h after treatment with different concentrations of PSMδ (0, 1, 3, 5, 7, 9 μM). Scale bar = 100 μm.Data are presented as mean ± s.d. Statistical significance was determined using a two-tailed Mann-Whitney U test (P: *< 0.05; **< 0.01; ***< 0.001; ****< 0.0001).

We evaluated the effects of *Staphylococcus epidermidis*-derived phenol-soluble modulins (PSMs) on human dermal papilla cell (HDPC) proliferation using the CCK-8 assay over 72 hours (**Figure 5A–L**).

PSMγ and PSMδ exhibited distinct time- and concentration-dependent profiles. Both peptides stimulated HDPC proliferation in a biphasic manner, promoting growth at lower concentrations while suppressing cell viability at higher doses. For PSMγ, no significant pro−proliferative effect was observed at 12 h at any concentration, and complete loss of viability occurred at 64–128 μM (**Figure 4A**). At 24 h, proliferation was significantly increased at 4 μM (*P* < 0.05), representing the peak effect, whereas concentrations ≥ 32 μM markedly suppressed proliferation (*P* < 0.001; **Figure 5B**). At 48 h, the effective concentration shifted downward to 2 μM (*P* < 0.05), and toxicity emerged at 16 μM (**Figure 5C**). By 72 h, no significant pro-proliferative effect was observed at any concentration, and marked toxicity was evident at ≥ 16 μM (**Figure 5D**).

PSMδ demonstrated a broader effective concentration range and more sustained activity. At 12 h, no significant difference from the control was observed at 1–32 μM, whereas 64–128 μM caused complete loss of viability (**Figure 5E**). At 24 h, proliferation was significantly increased at 2 μM (*P* < 0.05), with highly significant effects at 4–16 μM, and toxicity appearing at 32 μM (**Figure 5F**). At 48 h, significant pro−proliferative activity was already evident at 1 μM (P < 0.05), peaked at 2–8 μM, and remained significant at 16 μM before declining (**Figure 5G**). At 72 h, PSMδ was the only peptide retaining significant pro−proliferative activity, which was observed at 2–4 μM (*P* < 0.05), whereas 16 μM induced toxicity (**Figure 5H**).

Head−to−head comparison at matched concentrations and time points confirmed the superior potency of PSMδ (**Figures 5I–L**). At 12 h, no significant differences were observed between PSMγ− and PSMδ−treated cells at any corresponding concentration (**Figure 5I**). At 24 h, PSMγ elicited significantly stronger proliferation than PSMδ at 4 μM (*P* < 0.05), whereas PSMδ was significantly more potent than PSMγ at 16 μM (*P* < 0.0001; **Figure 5J**). At 48 h, PSMδ promoted proliferation to a significantly greater extent than PSMγ across the 1–8 μM range (*P* < 0.05; **Figure 5K**). By 72 h, only PSMδ at 2–4 μM maintained a pro−proliferative effect; PSMγ no longer exhibited any effective concentration range (**Figure 5L**).

### 3.10 PSMδ promotes HDPC migration in a concentration-dependent manner

**Figure 6:**
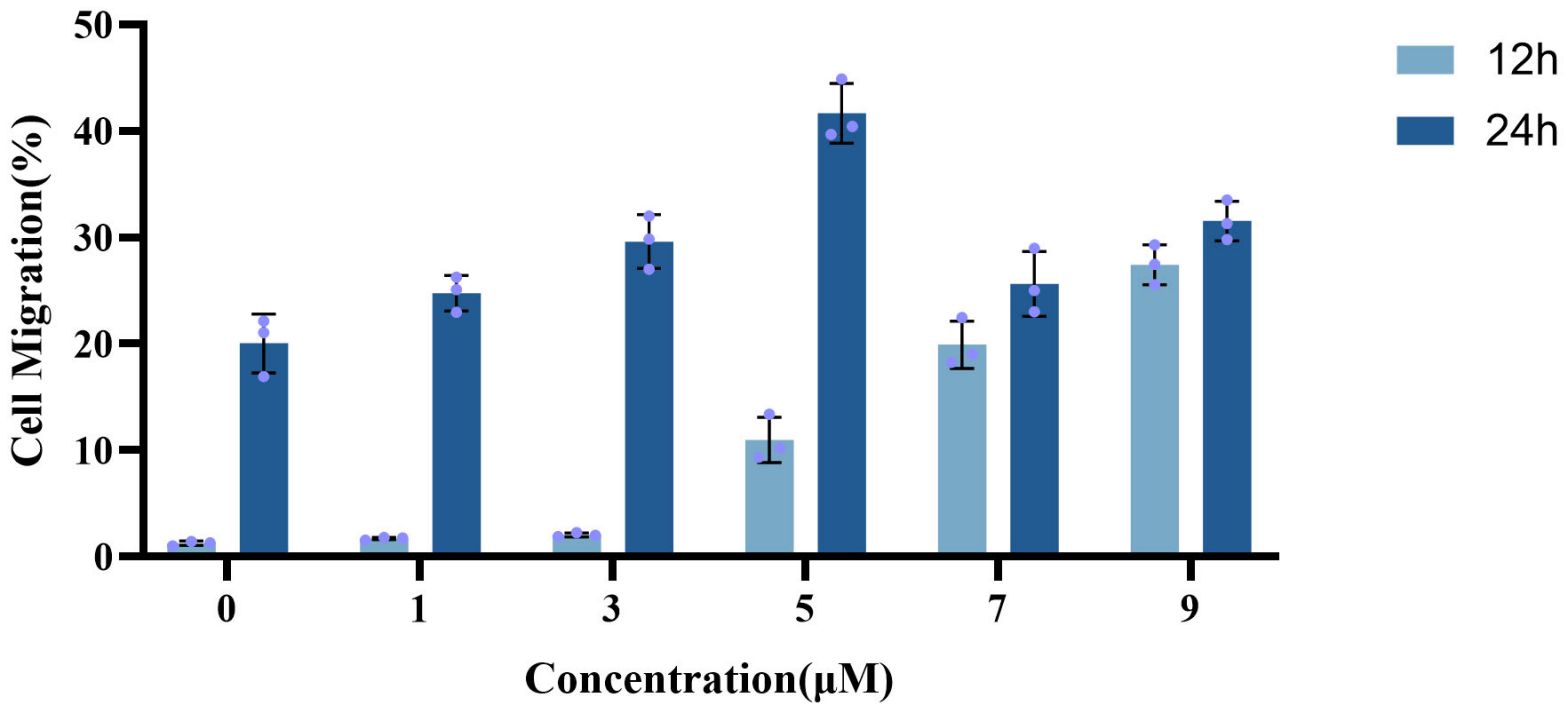
Quantitative analysis of PSMδ-induced HDPC migration. Migration rates of HDPCs at 12 h and 24 h after treatment with different concentrations of PSMδ (0–9 μM). Data are mean ± s.d. (n = 3). Statistical significance was determined by two-tailed Mann-Whitney U test. At 12 h, significant promotion of migration was observed at 5, 7, and 9 μM compared with control (all *P* < 0.01), with the highest rate at 9 μM (P < 0.05 vs. other concentrations). At 24 h, migration rates at 1 and 3 μM were significantly elevated relative to control (*P* < 0.05); the peak effect was achieved at 5 μM (*P* < 0.01), and 7 and 9 μM still showed significant pro-migratory effects compared with control (*P* < 0.05) but were significantly lower than at 5 μM (*P* < 0.01).

Based on the superior and more sustained pro-proliferative activity of PSMδ, we further evaluated its effect on HDPC migration using the scratch wound healing assay (**Figure 5M**). At 12 h, migration rates at 1 and 3 μM did not differ significantly from the control (*P* > 0.05), whereas 5, 7, and 9 μM significantly promoted migration (all *P* < 0.01), with the highest rate observed at 9 μM (*P* < 0.05). At 24 h, migration rates at 1 and 3 μM were significantly elevated relative to the control (*P* < 0.05). The migration rate peaked at 5 μM (*P* < 0.01), while higher concentrations (7 and 9 μM) showed reduced but still significant pro-migratory effects compared with the control (*P* < 0.05). These results demonstrate that PSMδ promotes HDPC migration in a concentration-dependent manner, with an optimal concentration of 9 μM at 12 h and 5 μM at 24 h under the tested conditions (**Figure 6**).

## 4 Discussion

This study, through a fine-scale comparative analysis of bacterial microbiota across four anatomical scalp regions in healthy individuals and AGA patients, reveals the regional heterogeneity of the scalp microbiome, providing experimental evidence for elucidating the spatial distribution patterns of AGA-associated microecological alterations.

### 4.1 Restructuring patterns of the core microbiota

Our findings confirm that *Cutibacterium* and *Staphylococcus* dominate the scalp bacteriome regardless of hair status, consistent with previous studies on the scalp microbiome (Shah et al., 2024). However, the patterns of change diverged markedly between the two groups. *Cutibacterium* showed no statistically significant difference at either the whole-scalp level or any individual region, displaying only marginal, directionally inconsistent fluctuations—a slight elevation in the frontal region and mild decreases elsewhere—suggesting that its abundance variations are likely driven more by local microenvironmental factors (e.g., sebum secretion, oxygen tension) than by the AGA disease state itself.

In striking contrast, *Staphylococcus* exhibited a significant reduction at the whole-scalp level in AGA patients, with this depletion being highly region-selective: it was most pronounced in the frontal region, followed by the vertex region, whereas the temporal and occipital regions showed only mild, statistically non-significant decreases. This anterior scalp-predominant pattern closely parallels the typical progression of AGA, implicating the loss of *Staphylococcus* as a critical accompanying event in AGA pathogenesis. As an important commensal genus within the hair follicle microecosystem, *Staphylococcus* can inhibit the overgrowth of opportunistic pathogens and modulate local immune homeostasis through the secretion of bacteriocins, lipoteichoic acid, and other molecules (Wang et al., 2021).

Moreover, an antagonistic relationship between *Cutibacterium* and *Staphylococcus* is well recognized (Nakatsuji et al., 2017; Wang et al., 2014; Nakamura et al., 2020). The slight (albeit statistically non-significant) increase of *Cutibacterium* in the frontal scalp of AGA patients may further suppress local *Staphylococcus* abundance through mechanisms such as metabolic competition, thereby rendering the *Staphylococcus* reduction most significant in the frontal region. Conversely, in the temporal and occipital regions, where *Cutibacterium* abundance was slightly lower, the inhibitory pressure on *Staphylococcus* was comparatively weaker, which may partially explain the less pronounced decline of *Staphylococcus in* these areas. Nevertheless, this microbial cross-talk hypothesis requires validation through longitudinal tracking or interventional experiments.

### 4.2 Elevated diversity suggests pathological microecological remodeling

Our diversity analyses revealed a notable community structural feature: AGA patients exhibited significantly higher species richness and community diversity at the whole-scalp level compared with healthy controls, whereas the Simpson index, which reflects the dominance concentration, showed a slight but statistically non-significant decrease. Regional trends were consistent with the whole-scalp pattern, albeit with varying magnitudes. This profile differs markedly from the microbial diversity loss and single-pathogen overgrowth commonly observed in inflammatory skin diseases such as atopic dermatitis (Gallo and Horswill, 2024) and acne (Podwojniak et al., 2025; O'Neill and Gallo, 2018), suggesting that AGA-associated scalp dysbiosis is not simply a matter of microbial pauperization or proportional imbalance but rather a pathological remodeling of community structure.

In conjunction with the altered core taxa, a potential mechanism underlying this elevated diversity may be that the marked reduction of dominant commensals such as *Staphylococcus* weakens the competitive exclusion exerted by the original resident microbiota, thereby creating opportunities for previously suppressed low-abundance taxa to colonize and expand. However, this increase in diversity does not signify a restoration of microbial homeostasis; instead, it more likely represents a passive community restructuring following the breakdown of the commensal barrier, which may further aggravate follicular damage by introducing potential opportunists or persistently perturbing the local immune microenvironment. β-diversity analysis revealed a significant, albeit modest, difference in community structure between groups, with the AGA group displaying lower within-group dispersion than the healthy group, suggesting a convergence and homogenization of the microbial community under the disease state—a finding that supports the inference that AGA exerts a directional influence on the scalp microbiome.

### 4.3 Animal model validation of the association between microbial dysbiosis and AGA

To validate the clinical findings under controlled experimental conditions, we employed a testosterone propionate-induced C57BL/6 mouse model of AGA to systematically evaluate alterations in the skin microbiota and their relationship with histopathology. Histological analysis demonstrated epidermal thinning, reduced hair follicle density with miniaturization, dermal thinning with disorganized collagen fibers, and immunofluorescence confirmed significant infiltration of macrophages and T lymphocytes, indicating that the model successfully recapitulated key features of human AGA.

At the microbial level, AGA mice displayed a profound restructuring of the skin microbiota. Notably, in stark contrast to the depletion of *Staphylococcus* observed in human AGA patients, *Staphylococcus* exhibited marked enrichment in the dorsal skin of AGA mice. We propose that this seemingly contradictory result actually reflects the differential presentation of the same pathological process across different time windows and host backgrounds, rather than a genuine conflict.

We suggest that this directional divergence reflects fundamentally different pathophysiological states and disease stages. Human AGA is a chronic, slowly progressive process: androgen levels rise gradually over years, sebaceous gland function declines progressively, and hair follicles miniaturize insidiously. This protracted deterioration of the microenvironment leads to the gradual replacement of *Staphylococcus*, which depends on sebum and intact follicular structures for colonization, by other taxa. In contrast, the mouse model represents an acute induction system: exogenous testosterone propionate injection causes a sharp surge in androgen levels, and hair follicles are synchronously driven into the anagen phase, creating a transient but permissive ecological niche for rapid *Staphylococcus* proliferation. Concurrently, the inherent Th1-type immune bias of C57BL/6 mice (Watanabe et al., 2004) maintains *Staphylococcus* at a very low baseline under healthy conditions; acute androgen challenge may temporarily weaken this immune surveillance, releasing *Staphylococcus* from suppression and triggering a marked rebound. Therefore, what the mouse model captures is likely the very early, transient “reactive commensal bloom” stage of human AGA that is difficult to detect in cross-sectional clinical samples.

We further propose that the reduction of *Staphylococcus* in human AGA may not be a homogeneous process, but rather a dynamic succession of species within this genus—a shift in the relative proportions of beneficial commensals and potential pathogens. We hypothesize that this succession may occur in three stages. In early-stage AGA, the initial alterations of the follicular microenvironment may act as a stress signal that transiently stimulates a reactive enrichment of commensal *staphylococci*, which attempt to maintain microecological homeostasis through secreted antimicrobial peptides and immunomodulatory factors. As the disease progresses to the intermediate stage, ongoing follicular miniaturization and altered sebum secretion progressively disrupt the colonization niche for commensals, leading to the gradual loss of beneficial species such as *S. epidermidis* and the concomitant weakening of the natural antagonistic barrier against pathogens. In late-stage, severely bald areas, the markedly compromised scalp barrier and dysregulated local immune surveillance may create a permissive environment for the overgrowth of pathogenic bacteria such as *S. aureus*; any detectable “rebound” of *Staphylococcus* at this stage may no longer signify the recovery of protective flora but rather the expansion of pathogenic strains.

This hypothesis resonates with the bidirectional cross-species discrepancy observed in our study: the acute *Staphylococcus* expansion in the mouse model may mirror the very early-stage reactive commensal bloom in human AGA, whereas the profound *Staphylococcus* depletion in the frontal scalp of chronic human AGA patients may correspond to the intermediate-to-late stage loss of commensals. Given that *16S* rRNA amplicon sequencing lacks the species-level resolution to reliably discriminate between *S. epidermidis* and *S. aureus*, this successional hypothesis at the species level awaits validation by metagenomic sequencing or species-specific quantitative assays.

Accordingly, the acute *Staphylococcus* expansion in the mouse model and the profound *Staphylococcus* depletion in the frontal scalp of human AGA patients can be reconciled within a unified successional framework: the former corresponds to the reactive commensal bloom of the early stage, and the latter corresponds to the commensal loss of the intermediate stage.

It is worth noting that, given the inability of *16S* rRNA amplicon sequencing to reliably distinguish between *S. epidermidis* and *S. aureus* at the species level, the above species-level successional hypothesis remains to be further validated. To this end, we plan to undertake the following studies in future research: (1) performing metagenomic shotgun sequencing or species-specific qPCR on existing human samples to delineate species-level shifts; (2) conducting a longitudinal observational study based on the successfully established mouse model, with continuous collection of skin microbial samples at multiple time points (e.g., days 0, 7, 14, and 30 after successful modeling) for sequencing, to track whether *Staphylococcus* abundance gradually declines or even shifts to depletion after the acute bloom, as predicted by the three-stage hypothesis; concurrently, collecting scalp samples from human AGA patients stratified by Hamilton-Norwood grade and performing shotgun metagenomic sequencing, so as to directly observe the dynamic changes of the microbial community across different disease stages and systematically compare disease progression with changes in microbial abundance; (3) establishing the causal relationship between *Staphylococcus* dynamics and hair follicle pathology through gnotobiotic or antibiotic-intervention experiments.

Therefore, despite the opposite directionality, the human and mouse findings converge on a core conclusion: *Staphylococcus* is highly sensitive to the AGA pathological state, and its significant fluctuation-whether a decrease or an increase-may serve as a sensitive bio-indicator of the pathological state. This cross-species differential fluctuation further supports the central logic of our study: the profound microbial community alterations are more likely a consequence secondary to the initiation of the AGA pathological process, rather than its initial trigger. The temporal sequence in the mouse model-testosterone propionate injection preceding microbial restructuring-provides experimental clues for distinguishing the “initiating cause” from the “aggravating factor.” However, this does not exclude the possibility that, once established, microbial abnormalities could participate in a positive feedback loop through pathways such as inflammatory activation, acting as a “secondary driver” that exacerbates follicular damage-a hypothesis whose verification is one of the core objectives of the aforementioned future studies.

### 4.4 PSMδ: a candidate molecule with dual potential to promote hair follicle cell activity and modulate the microecosystem

The selection of PSMγ and PSMδ for functional validation in this study was driven by our initial finding that *Staphylococcus* is a characteristically fluctuating genus in the AGA scalp microecosystem. Literature review further revealed that *S. epidermidis*, a prominent member of this genus, can serve as a beneficial commensal under certain conditions, and that PSMγ and PSMδ are representative phenol-soluble modulins secreted by this species with known antimicrobial and immunomodulatory functions (Cogen et al., 2010). However, existing research has primarily focused on their role in microbial community regulation, while their direct biological effects on host cells have remained unexplored. Given the intimate spatial relationship between *Staphylococcus* and the hair follicle microenvironment, we hypothesized that *S. epidermidis*-derived PSMs might directly act on hair follicle cells, thereby influencing hair growth. To test this, we exposed human dermal papilla cells (HDPCs) to PSMγ and PSMδ and assessed their proliferation and migration.

Our results demonstrate that both peptides regulate HDPC proliferation in a time- and concentration-dependent manner, yet their pharmacodynamic profiles differ markedly. PSMγ exhibits a narrow effective concentration window, transient action, and early emergence of cytotoxicity, whereas PSMδ maintains pro-proliferative effects over a broader concentration range with a more sustained duration of action. PSMδ also promotes HDPC migration in a concentration-dependent fashion.

These findings expand the functional paradigm of PSMs: they are not only effector molecules in microbial community competition but also direct regulators of host hair follicle cell biology. More importantly, when combined with the known ability of PSMδ to selectively kill pathogenic bacteria such as *S. aureus* while preserving the homeostatic colonization of commensals (Cogen et al., 2010), PSMδ emerges as a molecule with the dual potential to both modulate hair follicle cell activity and maintain microecological balance. This unique combination of attributes is not shared by existing hair−growth−promoting agents, which primarily target hair follicle cells or androgen signaling pathways, largely overlooking the regulation of the follicular microenvironment. The discovery of PSMδ thus provides preliminary experimental evidence and a proof−of−concept for developing novel intervention strategies from the perspective of “microecosystem–hair follicle” interactions. Nevertheless, its *in vivo* release kinetics, local concentration maintenance, and long−term safety remain critical challenges for future investigation.

### 4.5 Limitations

This study, from the perspective of the scalp microecosystem, has revealed that microbial dysbiosis may participate in the development and progression of AGA, preliminarily elucidated the key significance of aberrant *Staphylococcus* abundance fluctuations and the potential function of PSMδ, and provided new experimental evidence and research directions for microbiome-targeted intervention in AGA. However, several limitations should be acknowledged. First, the clinical sample size was relatively modest (12 AGA patients and 12 controls, yielding 96 scalp microbial samples in total), and to minimize the confounding effects of hormonal and other factors, only male subjects were enrolled. Second, 16S rRNA amplicon sequencing lacks the species-level resolution required to reliably distinguish between closely related species such as *S. epidermidis* and *S. aureus*. This limitation precluded an in-depth dissection of the cross-species divergence in *Staphylococcus* abundance trends between the human and mouse models, and the three-phase species succession hypothesis proposed herein therefore awaits validation by metagenomic sequencing or species-specific quantitative assays. Third, this study strategically focused on the bacterial compartment and did not include fungal community profiling. Given that the role of fungi such as *Malassezia* in the AGA scalp microecosystem has been relatively well-documented, future integration of fungal ITS sequencing or metagenomic sequencing will help construct a more complete multi-kingdom scalp microbial atlas and explore the potential contribution of bacterial–fungal interactions to scalp microecosystem imbalance. Fourth, this study only preliminarily explored the effects of PSMδ on human dermal papilla cell proliferation and migration, without involving more in-depth mechanistic validation such as signaling pathway dissection or *in vivo* functional experiments. Fifth, the study as a whole remains at an exploratory stage, and interventional experiments and clinical translational studies have not yet been conducted. Future studies will further expand the sample size and include female patients, employ metagenomic sequencing and longitudinal designs to deeply dissect species-level microbial succession patterns, integrate fungal community analysis to construct multi-kingdom microbial interaction networks, systematically validate the mechanism of action of PSMδ through *in vivo* models and signaling pathway studies, and ultimately advance the clinical translation of microecosystem-targeted strategies.

## Data Availability

The high-throughput sequence data that support the findings of this study have been deposited in the National Centre for Biotechnology Information (NCBI) BioProject database under the project numbers PRJNA1478247 (https://www.ncbi.nlm.nih.gov/bioproject/PRJNA1478247) and PRJNA1478412 (https://www.ncbi.nlm.nih.gov/bioproject/PRJNA1478412), and will be made publicly available on publication or on request during peer review.
