# Supplementary material for: Scalp microbiome in male androgenetic alopecia: 16S rRNA sequencing-based clinical characterization, mouse model validation, and effects on hair follicle cells
Source: Front Cell Infect Microbiol. 2026 Jul 1;16:1878609. doi: 10.3389/fcimb.2026.1878609 (PMC13368710; doi:10.3389/fcimb.2026.1878609)
Supplement: Supplementary file 1 [file Table1.docx]

Supplementary Material

**Supplementary Material 1:Diagnostic Criteria and Differential Diagnoses for AGA**

Supplementary Methods: Diagnostic Criteria and Differential Diagnoses for Androgenetic Alopecia

Diagnostic criteria (based on the Chinese Guidelines for the Diagnosis and Treatment of Androgenetic Alopecia [2023])

1. Core clinical criteria

• Onset during or after puberty; progressive, chronic course (≥ 6 months); no acute massive shedding.

• Typical male pattern hair loss: frontal hairline recession and/or vertex thinning with progressive hair shaft miniaturization.

• Family history consistent with autosomal dominant inheritance (primarily paternal).

• Exclusion of cicatricial alopecias: no scarring, no significant erythema, inflammation, or pain on the scalp.

2. Auxiliary examinations

• Dermoscopy (key diagnostic tool): hair shaft diameter heterogeneity (anisomastia) > 20% is the core sign, with mixed terminal and vellus hairs; may also show increased vellus hair ratio, increased single‑hair follicular units, peripilar signs, focal atrichia, yellow dots, and scalp hyperpigmentation.

• Hair pull test: normally negative in AGA (positive indicates active shedding, common in telogen effluvium and alopecia areata).

3. Differential diagnoses

• Telogen effluvium: acute onset, often with a clear trigger; diffuse hair loss; positive pull test; dermoscopy shows empty follicles and upright regrowing hairs without marked miniaturization.

• Diffuse alopecia areata: acute onset; positive pull test; hair loss not limited to fronto‑vertical areas; dermoscopy shows exclamation‑mark hairs and black dots.

• Drug‑induced alopecia: clear medication history; mostly diffuse hair loss; improvement upon drug cessation.

• Frontal fibrosing alopecia: band‑like frontal hairline recession with skin atrophy; a form of cicatricial alopecia.

• Fibrosing alopecia in a pattern distribution: features of both lichen planopilaris and pattern‑distribution hair loss; diagnosed by dermoscopy and histopathology.

**Supplementary Methods 2: Full Inclusion/Exclusion Criteria**

**AGA Patient Group**

**Inclusion criteria:**

• Male, aged 18–40 years.

• BMI 18.5–24.0 kg/m².

• Diagnosed with AGA according to the Chinese Guidelines for the Diagnosis and Treatment of Androgenetic Alopecia (2023), with Hamilton–Norwood grade II–V.

• No history of scalp perming or dyeing within 12 months prior to sampling.

• No use of anti‑tumor drugs, immunosuppressants, or radiotherapy within 3 months prior to sampling.

• No use of drugs or functional shampoos that could affect scalp microecology or hair growth within 3 months (including but not limited to antibiotics, antifungals, corticosteroids, 5α‑reductase inhibitors, minoxidil, retinoids, and medicated shampoos containing ketoconazole or selenium sulfide).

• No systemic antibiotics within 30 days prior to sampling.

• No probiotics within 15 days prior to sampling.

• Last routine hair wash completed 48 hours before sampling with no further washing until sample collection.

• No history of any scalp disease (seborrheic dermatitis, psoriasis, folliculitis, cicatricial alopecia, alopecia areata, etc.).

• Willingness to participate and provision of written informed consent.

**Exclusion criteria:**

• Hamilton–Norwood grade I, VI, or VII.

• Presence of any scalp disease or injury (seborrheic dermatitis, psoriasis, folliculitis, alopecia areata, frontal fibrosing alopecia, fibrosing alopecia in a pattern distribution, etc.).

• Use of prohibited medications or shampoos listed in the inclusion criteria within 3 months prior to enrollment.

• Use of systemic antibiotics within 30 days or probiotics within 15 days prior to enrollment.

• Anti-tumor, immunosuppressive, or radiotherapy within 3 months prior to enrollment.

• Severe systemic diseases (cardiovascular, hepatic, renal, endocrine, hematological disorders, malignancies, or immunodeficiency).

• BMI < 18.5 kg/m² or > 24.0 kg/m².

• Scalp perming or dyeing within 12 months prior to enrollment.

• Failure to comply with the pre-sampling washing requirement (last wash 48 hours before sampling).

• Major psychological stress, significant weight loss, or unhealthy lifestyle habits (alcohol abuse, smoking ≥ 10 cigarettes/day for ≥ 1 year) within 3 months prior to enrollment.

• Concurrent participation in other clinical studies.

• Use of anti-androgen or minoxidil therapy within 3 months prior to enrollment.

• Unwillingness to participate or poor compliance.

**Healthy Control Group**

**Inclusion criteria:**

• Male, aged 18–40 years, comparable to the AGA group in age and sex composition.

• BMI 18.5–24.0 kg/m².

• Confirmed free of any dermatological, scalp, or systemic diseases by clinical examination, with normal scalp appearance and hair status.

• No history of scalp perming or dyeing within 12 months prior to sampling.

• No use of anti‑tumor drugs, immunosuppressants, or radiotherapy within 3 months prior to sampling.

• No use of drugs or functional shampoos that could affect scalp microecology or hair growth within 3 months (same scope as the AGA group).

• No systemic antibiotics within 30 days prior to sampling.

• No probiotics within 15 days prior to sampling.

• Last routine hair wash completed 48 hours before sampling with no further washing until sample collection.

• No history of any scalp disease (seborrheic dermatitis, psoriasis, folliculitis, cicatricial alopecia, alopecia areata, etc.).

• Willingness to participate and provision of written informed consent.

**Exclusion criteria:**

• Meeting the diagnostic criteria for AGA according to the Chinese Guidelines (2023), or presence of any form of pathological hair loss (including but not limited to alopecia areata, telogen effluvium, cicatricial alopecia, etc.).

• Presence of any scalp disease or injury (seborrheic dermatitis, psoriasis, folliculitis, fungal infection of the scalp, etc.).

• Use of prohibited medications or shampoos listed in the inclusion criteria within 3 months prior to enrollment.

• Use of systemic antibiotics within 30 days or probiotics within 15 days prior to enrollment.

• Anti-tumor, immunosuppressive, or radiotherapy within 3 months prior to enrollment.

• Severe systemic diseases (cardiovascular, hepatic, renal, endocrine, hematological disorders, malignancies, or immunodeficiency).

• BMI < 18.5 kg/m² or > 24.0 kg/m².

• Scalp perming or dyeing within 12 months prior to enrollment.

• Failure to comply with the pre‑sampling washing requirement (last wash 48 hours before sampling).

• Major psychological stress, significant weight loss, or unhealthy lifestyle habits (alcohol abuse, smoking ≥ 10 cigarettes/day for ≥ 1 year) within 3 months prior to enrollment.

• Concurrent participation in other clinical studies.

• Unwillingness to participate or poor compliance.
